# Pre-trained artificial intelligence-aided analysis of nanoparticles using the segment anything model

**DOI:** 10.1038/s41598-025-86327-x

**Published:** 2025-01-17

**Authors:** Gabriel A. A. Monteiro, Bruno A. A. Monteiro, Jefersson A. dos Santos, Alexander Wittemann

**Affiliations:** 1 Colloid Chemistry, Department of Chemistry, University of Konstanz, Universitaetsstrasse 10, 78464 Konstanz, Germany; 2 Pattern Recognition and Earth Observation Laboratory, Department of Computer Science, Federal University of Minas Gerais, Belo Horizonte, 31270-901 Brazil; 3 Department of Computer Science, University of Sheffield, S1 4DP Sheffield, UK

**Keywords:** Microscopy, Image processing, Nanoparticles, Artificial intelligence, Segment anything model, Particle morphology, Colloids, Imaging studies, Microscopy, Scanning electron microscopy, Characterization and analytical techniques, Nanoparticles, Microscopy, Scanning electron microscopy, Characterization and analytical techniques, Scientific data, Computational science

## Abstract

Complex structures can be understood as compositions of smaller, more basic elements. The characterization of these structures requires an analysis of their constituents and their spatial configuration. Examples can be found in systems as diverse as galaxies, alloys, living tissues, cells, and even nanoparticles. In the latter field, the most challenging examples are those of subdivided particles and particle-based materials, due to the close proximity of their constituents. The characterization of such nanostructured materials is typically conducted through the utilization of micrographs. Despite the importance of micrograph analysis, the extraction of quantitative data is often constrained. The presented effort demonstrates the morphological characterization of subdivided particles utilizing a pre-trained artificial intelligence model. The results are validated using three types of nanoparticles: nanospheres, dumbbells, and trimers. The automated segmentation of whole particles, as well as their individual subdivisions, is investigated using the Segment Anything Model, which is based on a pre-trained neural network. The subdivisions of the particles are organized into sets, which presents a novel approach in this field. These sets collate data derived from a large ensemble of specific particle domains indicating to which particle each subdomain belongs. The arrangement of subdivisions into sets to characterize complex nanoparticles expands the information gathered from microscopy analysis. The presented method, which employs a pre-trained deep learning model, outperforms traditional techniques by circumventing systemic errors and human bias. It can effectively automate the analysis of particles, thereby providing more accurate and efficient results.

## Introduction

### Nanoparticles and their morphological characterization

The study of colloidal particles concerns the analysis of mixtures comprising discontinuous domains, which are randomly distributed in a continuous phase^1^. This essentially means that a large amount (sometimes over 10^15^ elements per mL) of very small particles (usually in the range of nm to $\mu$m) is present^2^. These colloidal particles can be used in a wide array of applications encompassing coatings^3^, adhesives^4^, lubricants and biolubricants^5^, catalysts^6^ and catalyst membranes^7^, rheology modifiers^8^, pharmaceutical^4^, and food products^9^. In the academic context, model colloids are crucial for the understanding of important physical and physico-chemical phenomena like sedimentation^10^ and crystallization^11^. This encompasses the study of nucleation and growth processes^12,13^. The understanding gathered from studies using model colloids can then be employed in the development of colloidal molecules^14,15^, colloidal crystals^11^, and self-assembled superstructures^12,13^. Since particle morphology plays a decisive role in the behavior of colloidal systems, it is essential to conduct a comprehensive morphological characterization of any given sample^12^. This analysis is commonly done using micrographs, either obtained from optical microscopy^16^, scanning electron microscopy (SEM), transmission electron microscopy (TEM)^17,18^, or atomic force microscopy (AFM)^18^. Regardless of how the images are obtained, it is customary to measure a minimum of 500 particles in order to obtain statistically relevant results^19^. This process is inherently time-consuming and prone to human bias and systemic errors if conducted manually, thereby underscoring the necessity for the development of reliable automated measuring techniques.

Such techniques allow for the expansion of the information gathered from images, with applicability across a series of very distinct fields. These include the analysis of tumor tissue^20^, substructures in cells^21^, tracking of cellular activity^22^, defects in the structure of materials^23^, metal alloys^24^, particles, particle-based materials^25–29^, and particle tracking^30^. For particle aggregates, however, the available characterization methods still lack the capability to analyze the aggregates themselves and the particles that compose them simultaneously. This means that while the morphological characterization of a particle within a particle aggregate may be obtained, it cannot be obtained from the aggregate itself^27,28^. The converse is also evidenced in the literature, where the superstructure is analyzed from micrographs, but the particles within are not^12^. Characterizing the aggregates using the particles contained in them would allow for a deeper understanding of the morphology of the aggregate, particularly in terms of internal subdivisions they possess. The same is also valid for complex multi-lobed particles. In the latter case, a comprehensive morphological analysis necessitates not only the differentiation between particles but also between the segments within each particle.

Given that morphology is a defining factor in colloidal particles’ behavior, their correct morphological characterization is essential. Successful workflows for characterizing particles from micrographs use either Bayesian segmentation^27^ or neural networks (NN)^28^. The former is more widely employed for colloidal particles^26,31^. Irrespective of the strategy used to extract morphological data from the images, this information must allow for determining parameters such as particle size and particle size distribution. The segmentation based on traditional methodologies may produce inaccurate segmentation labels and fails to identify regions comprising more than one element. Such issues may be addressed through the utilization of more contemporary approaches, such as machine learning^29^ and deep learning methodologies^32^.

### Algorithmic approaches in image analysis

Computer vision is a field of artificial intelligence (AI) concerned with the extraction of information from visual inputs, such as digital images or videos. Among the applications of computer vision, the semantic segmentation of images is worthy of particular mention, as it deals with the differentiation of elements present in a given image. In essence, semantic segmentation entails the labeling of each class of pixels or region in an image (or video) with a semantic meaning^33^. In the specific case under discussion, semantic segmentation can be defined as the process of classifying the pixels in a micrograph as either belonging to a given particle or not. This results in the creation of matrices known as masks, which represent the projection of a segmented area in an image. Several algorithms have been proposed to handle this task, such as thresholding^34^, watershed transformations^35^, quick shift algorithm^36^, simple linear iterative clustering (SLIC)^37^, and also, “shallow” machine learning algorithms, like random forest^38,39^. However, these AI approaches, in the form of shallow machine learning, are frequently limited in their capacity to analyze raw data input. This incapacity stems from the fact that the shallow models only incorporate one level of data transformation, which limits their data processing efficiency^38,39^.

One of the most frequently employed metrics for evaluating the precision of computer vision tasks is the comparison of a new method with a *ground truth* segmentation. A result that is closer to the *ground truth* is considered to be of higher quality than one that deviates from this value. In some cases, the *ground truth* must be provided by specialists who analyze the particles manually, which is also a challenging process. Despite the indication of accuracy provided by comparing the automated measurements to the *ground truth*, it is not inherently necessary to attest to the reproducibility of the developed technique^40^.

As a branch of machine learning, deep learning^32^ has been further developed over the past few decades, enabling the identification of more intricate correlations than before. The high complexity of the analyses made possible by deep learning has led to these models becoming a significant component in pattern recognition tasks, such as image classification^41^, object detection and instance segmentation^42^, and semantic segmentation^43,44^. This adoption has occurred in a plethora of areas, including medical images^45^, autonomous driving^46^, remote sensing^47^, seismic interpretation^48^, and on the aforementioned analysis of microscopy images of colloidal particles^28,30^. The efficacy of these models relies on the multi-level representations of raw data *via* non-linear transformations, thereby enabling the encoding of semantic representations that can be leveraged for pattern recognition^32^. Nevertheless, the performance of deep learning models is highly correlated to the amount and quality of data available during the training phase^49^. The process of labeling data can be a slow, time-consuming, and biased endeavor. Consequently, there is a growing interest to generalize to tasks where only a few images are available for training^50^. These settings are referred to as few-shot scenarios^51^ or zero-shot^52^. The zero-shot scenario is an extreme case of generalization, whereby a pre-trained model is deployed in a target domain, for which it was not specifically trained. This circumvents the shortcomings associated with the necessity of training a model while retaining the capacity for semantic segmentation-related operations.

### Segment anything model

The Segment Anything Model (SAM)^53^ is a powerful method trained over a huge and diverse basis of annotated segmentation tasks. Thus, SAM is capable of achieving high-quality semantic segmentation results in several tasks without domain-specific pre-training. Recently, SAM^53^ has been proposed as a method capable of segmenting images without the need of further pre-training, applicable to a broad range of image types. This represents an innovative task and solution to zero-shot learning in certain scenarios that aim at segmenting any instances in an image without requiring further training of models. The straightforward usability of the SAM, along with its efficiency^53^, lends itself to effective analysis of intricate structures within micrographs. In the case of complex colloids, this segmentation routine substitutes the manual measurements that had already been performed for multi-lobed complex nanoparticles^2,19^, thereby saving time and minimizing the potential for human error^23,54^. To date, SAM has only been employed^55^ for the identification of particles without any accompanying morphological analysis or validation against experimental results. The absence of validated morphological analysis from SAM-generated segmented images shows that there is a lot of unexplored potential in the field of particle characterization from segmented images. Consequently, we aim to explore the relevant features that can be leveraged from it. By utilizing simple optimization techniques, it should be possible to identify both individual elements and allocate them into aggregates they belong to. This approach facilitates the morphological analysis for complex particles and also allow for the analysis of individual elements within these particles.

### Outline

We present an analysis routine based on a pre-trained deep learning model for image segmentation that allows determination of the dimensions and morphology of structures with different levels of complexity depicted in micrographs. The model particles differ in their morphological complexity and were chosen to confirm that the developed method can extract morphological information from complex arrangements. As a proof of concept, we analyze the particles and their subdivisions and extract morphological information that is only obtainable when these analyses are combined. The presented analyses dismiss the need to train a dedicated deep learning-based model. This was achieved by using a previously trained general segmentation model and optimizing post-processing techniques. The development presented here can expand and accelerate the existing capabilities of the methods whilst also making them more accurate. To validate the data obtained here, the measurements performed are compared to the measurements provided when the model particles were first presented^19^. The results show that a generalist segmentation model can reliably be used to analyze complex nanostructures and extract information present across multiple levels of organization with complete reliability. The analysis routine presented here also avoids the systemic errors associated with manually measuring complex particles.

## Methods

### Dataset

The preparation of spheres, dimers, and trimers was achieved using emulsion polymerization and a series of seeded emulsion polymerizations along the lines given recently^19^. All electron micrographs presented were obtained using field-emission scanning electron microscopy on a Gemini 500 microscope (Carl Zeiss A.G., Oberkochen, Germany) with an operation tension of 3kV. All images were taken at a magnification of 25,000 $\times$ and analyzed as received from the microscope.

### Manual measurements

A series of manual measurements were conducted as a means of establishing a benchmark, the *ground truth*, for the subsequent automated image analyses. The manual measurements were conducted utilizing the oval measuring tool in the ImageJ software (NIH, US Department of Health and Human Services, Washington, D.C., U.S.A.). ImageJ was selected for use in this process due to its broad applicability and prominent position in the field of image analysis^26,56^. Each mask was created manually and represented a circular entity. The longest line segment that can be fitted into the oval masks represents the diameter of the circular entity.

### Watershed method

The image analysis referred to as reference method (RM) was centered on the masking technique using the ImageJ software. The widespread use of the ImageJ software and the large community using it led to the publishing of extensive libraries of plugins for it. These plugins, which are also used here, expand the software’s capabilities beyond what it was originally intended for^56^. In order to measure the particles in this study using the ImageJ *Watershed* plugin, it was necessary to tune the plugin’s parameters, making the analysis presented here distinct from what is usually done with ImageJ. When treating and analyzing the images, the first plugins used were the *median filter* and the *rolling ball background subtraction* plugins. This process removed the background, leaving only the particles to be analyzed. These treated images were then converted to binary images using the *AutoThreshold* plugin. For all the samples, the *watershed* plugin was then used in a process that consisted of segmenting the images using a low-sensitivity transformation and then saving the data for the resulting masks. The same procedure was repeated for the spheres and the dumbbells, but now with a higher watershed transformation sensitivity. The methodology of using a higher sensitivity watershed transformation to promote the individual masking of the trimer lobes was also tested but failed to deliver consistent results (see the Supplementary Information section, Fig. SI 1 and 2). This led to the need to conduct the second round of measurements by selecting the oval measuring tool in the ImageJ software and *manually* fitting ellipses to each of the trimer lobes. Regardless of the analyzed sample, the masks obtained in the first step were used as references to assign the masks obtained in the second step to each other. This was done using a custom post-processing code based on comparing the position of the masks obtained in the two subsequent masking procedures. This ensures that the mask representing a given lobe is contained within the mask representing a given complete particle. For the spheres, only one mask obtained in the second masking process was observed for each mask obtained in the first masking step. This is to be expected since the spheres are not subdivided. For the dumbbells and trimers, assigning smaller masks representing the lobes to a larger one representing the entire particle allows for the determination of which lobes belong to each particle. This also makes it possible to determine which lobes belong to the same set and constitute the same multi-lobed particle. The post-processing stage serves to augment the array of information provided by the ImageJ software. The post-processing algorithm excludes instances with areas that are less than half the mean area of all masks for a given image. Furthermore, masks exceeding 1.5 times the mean area of all masks in a given image are rejected. This process is possible due to the use of model particles with minimal dispersity in this study^19^. Furthermore, the aspect ratio and circularity of the masks are employed as parameters for their rejection during post-processing. Any masks with an aspect ratio exceeding 1.15 and a circularity or roundness of less than 0.85 are not considered to be spheres. Similarly, any masks with an aspect ratio of less than 1.4 are not considered to be dimers. This is achieved by organizing the particles into sets using their relative positions. The details used during the implementation of this method are presented in the project’s GitHub and institutional servers linked in the *Additional Information* section. These details include the ImageJ macros and the post-processing algorithm developed to treat the data obtained from the macros.

### SAM-based method

The image segmentation for the SAM-based method was based on the automatic generation of masks using one single forward pass. The model’s parameters regarding the density of points sampled were adjusted for the particle size. The stability threshold was also adapted for the actual case to avoid duplicated and poor-quality masks. After the mask generation provided by the SAM, the segments were filtered to avoid the identification of foreign objects or areas of background as particles. Similarly, the circularity of the particles was also used as a filter to avoid unrelated masks. The mask features, such as diameter, area, perimeter, position, etc., were obtained using the *regionprops* algorithm from Scikit-Image^57^.

The SAM can segment between the lobes but does not directly provide the information that two or more lobes belong to the same set. This is not a problem when analyzing spherical particles, as they are not subdivided. This translates to the fact that, for the spheres, the described masking and measuring method constitutes the complete analysis. As no subdivisions exist in the spherical particles, each mask obtained is analyzed as a stand-alone element and is individually measured using the *regionsprops* algorithm. For the dumbbells and trimers, however, the masks representing each lobe must first be assigned to those representing the other lobes in the same particle. In this case, the assignment process was done by minimizing the distance between the centroids of the lobes. Assigning the lobe masks to the other lobes constituting the same particle expands what the SAM delivers. With this expansion, it is possible to measure the collective morphological properties of multi-lobed particles. These collective morphological properties, like for the RM, include the distance between the lobe centroids and the angle between the three trimer lobe centroids. The implementation details can be found in the project’s GitHub and institutional servers (see Accession codes in the Data Availability Section).

The utilization of the Segment Anything Model as a segmentation methodology also presents certain challenges that are addressed during post-processing. The primary factor is that the Model can provide overlapping masks, whereby a pixel can be assigned to more than one mask during the semantic segmentation. This issue was addressed through the development of a post-processing algorithm, which involved repeating the same requirements demonstrated for the post-processing of the watershed-based RM measurements.

## Results and discussion

**Figure 1:**
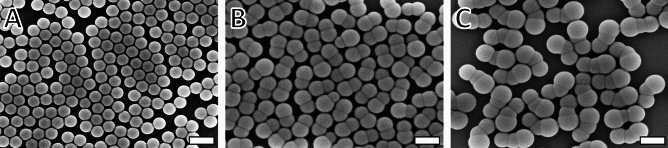
Micrographs of (**A**) nanospheres (SphPS), (**B**) two-lobed dumbbell nanoparticles (DiPS), and (C) and three-lobed trimer nanoparticles (TriPS) with scale bars representing 250 nm. Throughout this study, these three sets of particles were used as a proof of concept for the automated SAM-based analysis presented here.

Particle morphology is known to greatly influence the self-assembly and hydrodynamic properties of colloidal suspensions^15^. Given its vital importance, extensive morphological characterization is necessary for these particles^12,19^. A thorough morphological characterization based on image segmentation and subdivision recognition can also be conducted for particle-based materials^25^ and other large structures^20^. In either case, a rigorous understanding of the morphology is needed, including the ability to determine the size of the subdivisions and their relative positions. A range of sets of particles with increasing complexity was employed in this study to confirm the applicability of the method developed. The different sets are morphologically distinct and demand different kinds of post-processing. The nanospheres SphPS (Fig. 1A) are the simplest to analyze as they are not subdivided. Their measurement was used to confirm that the size of nanoparticles can be determined by processing segmented images and to match up-to-date image analysis techniques. The analysis of the dumbbell nanoparticles DiPS (Fig. 1B) demands data processing with higher complexity, as the particles are composed of two separate segments. In this case, the capability to measure structures with higher complexity was attested by determining the distance between the center of each particle lobe. The trimer nanoparticles TriPS (Fig. 1C) have a total of three subdivisions. This is displayed in a more complex analysis when compared to both the SphPS and DiPS nanoparticles. In the case of TriPS, the parameter used to validate that the collective morphology can be determined using the methodology developed here was the widest (i.e., central) angle between the centroids of the segments. The angle of the TriPS is an important morphological feature, as it determines attributes like the aspect ratio and the overall hydrodynamic behavior and self-assembly of the nanoparticles.

### Analysis of spherical particles

**Figure 2:**
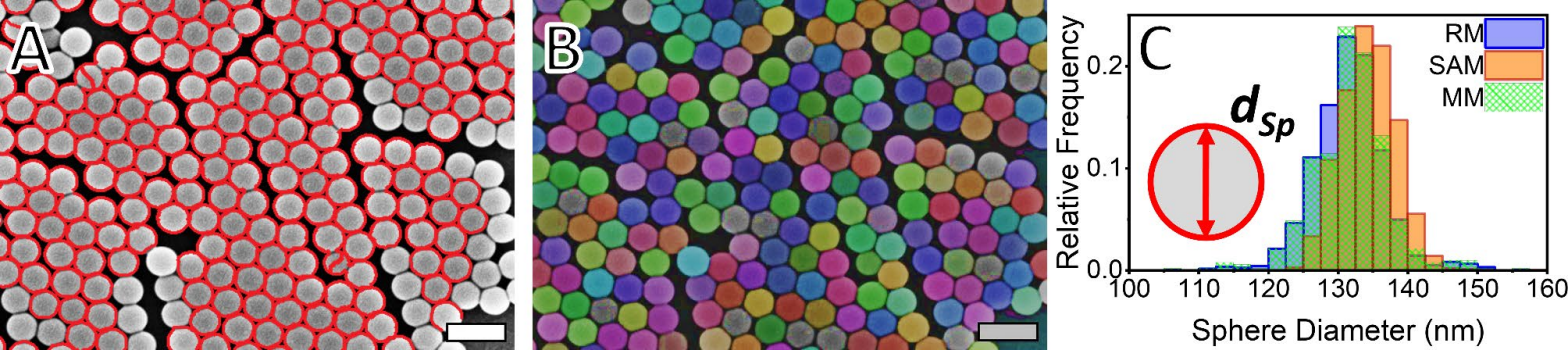
Comparison between the segmentation of the microscopy images performed using the (**A**) watershed-based RM and the (**B**) SAM-based method. (**C**) The distributions of sphere diameters for MM, RM, and SAM exhibit a high degree of coincidence with the MM, here taken as the *ground truth*. The fact that RM and SAM strongly coincide with the *ground truth* demonstrates the precision and reliability of both automated processes in determining the dimensions of elements within microscopy images. The scale bars represent 250 nm.

For the analysis of the SphPS nanoparticles, manual measurements were taken as a direct basis for comparison. In Fig. 2, the comparison between the segmentation techniques is shown. The same FESEM image is first presented as segmented by the RM (Fig. 2A) and by the SAM-based method (Fig. 2B). Initially, it is possible to observe that in both cases, the projections of the spheres in the micrographs are identified individually. Although the RM method was capable of identifying the spheres individually, some of the sphere projections (<1%) were segmented into two parts. For the same method, some particles were not identified and did not appear as masks, showing a tendency to not properly recognize the particles on the edge of an organized domain. These two segmentation errors were not observed for the SAM, where the particles were adequately segmented with no overlap. The fact that the spheres were not subdivided and that the spheres on the edges of organized domains were also identified by the SAM-based method shows an advantage of SAM over RM.

It should be noted, however, that these systemic errors in RM do not affect the size distribution. This can be ascertained by the exclusion of oversegmented masks from the analysis during post-processing. Nevertheless, the subdivision errors have no impact on the sphere diameter (*d*_Sp_) distributions illustrated in Fig. 2A. This is evidenced by the concordance between the RM and SAM measurements and the MM, the last representing the *ground truth* here. The high degree of agreement between the measurements demonstrates that both RM and SAM are effective in determining the diameter size distributions of spheres in micrographs with reliability. The RM method is based on the watershed algorithm, which has been optimized for use with circular masks^58,59^. In contrast, the SAM method has yet to be proven to be reliable for determining such distributions. From this point onwards, both the size distributions obtained with RM and with SAM can be considered to be accurate. This benchmark is important for attesting to the accuracy of the method and demonstrating that SAM can be applied to determine the size distribution as effectively as the more commonly used ImageJ^60^ and dedicated NNs^28^.

The agreement between the data observed for the size distributions (Fig. 2C) is also present when the mean diameter values (*d*_Sp,n_) are compared (Table 1). The results demonstrate that the *d*_Sp,n_ values obtained with RM and SAM exhibit a maximum discrepancy of less than 2% in comparison to the benchmark manual measurements. This difference lies within the standard deviation (SD) of the measured distributions, and is lower than the error of 5% expected for electron microscopy itself^17,18^. Despite the agreement in the data obtained using RM and SAM regarding *d*_Sp,n_ and SD, the polydispersity index (PDI) values of these distributions do vary. A PDI value of 1 represents a sample in which all the particles have the same size. The higher the PDI value is, the broader the size distribution of the sample is. When using the SAM, a lower dispersity of the particles is found than in the case of the RM. This reflects the narrower size distribution observed in Fig. 2C for SAM compared to RM.

**Table 1:** Comparison between the diameter distributions obtained from images segmented manually, using the Euclidean distance-based watershed methodology (RM) and the Segment Anything Model-based method (SAM).

Measurement	Manual measurements			Reference method			SAM-based method		
	Mean (nm)	SD (nm)	PDI	Mean (nm)	SD (nm)	PDI	Mean (nm)	SD (nm)	PDI
Sphere diameter	132	6	1.0018	132	5	1.0016	134	4	1.0009

The mean sphere diameters (*d*_Sp,n_) differ by less than 2%, a value lower than the error expected for measurements taken using electron microscopy. This observation reflects the high agreement between the *d*_Sp_ distributions obtained using manual measurements, RM and SAM. The distributions, however, are not rigorously the same, with the SAM-based method having a slightly lower polydispersity index (PDI), reflecting a narrower distribution.

### Analysis of dumbbell particles

The analysis of the dumbbell nanoparticles adds extra layers of complexity when compared to the analysis routine presented for the spheres. The extra challenge is characterized by the need to identify the dumbbell lobes as two entities that belong to the same set of particles. Conducting the analysis in such a manner is crucial, as some morphological and hydrodynamic aspects depend on both lobes and their relative positioning^10^. The unfulfilled need to extract morphological data from images with the described depth demonstrates that this kind of analysis is not trivial. This section describes the morphological characterization of two-lobed particles. Here, the lobes are identified as stand-alone elements and as constituents of the same set. This depth of identification allows for the determination of properties that are individual to each lobe (*i.e.* lobe diameter) and that apply to both lobes (*i.e.* distance between the center of the dumbbell lobes).

In the analysis of dumbbells, both RM and SAM have been demonstrated to be effective methods for determining diameter distributions. This consideration, which is rooted in the findings for spherical particles, is pivotal for the subsequent observations. A manual segmentation of the dumbbells would inevitably introduce systemic errors, rendering the data unusable for the determination of the distance between the dumbbell lobes. The systemic errors originate from the methodology employed to ascertain the distance between the dumbbell lobes. This is achieved by identifying their respective centers as their centroids. As the manual segmentation process would also entail the superimposition of the masks representing each dumbbell lobe, the distance between the centroids is consequently distorted to values that are less than the actual distance. Given the unavoidable presence of systemic errors associated with MM and the proven accuracy of RM and SAM, only the automated analyses were conducted in the further course of the study.

**Figure 3:**
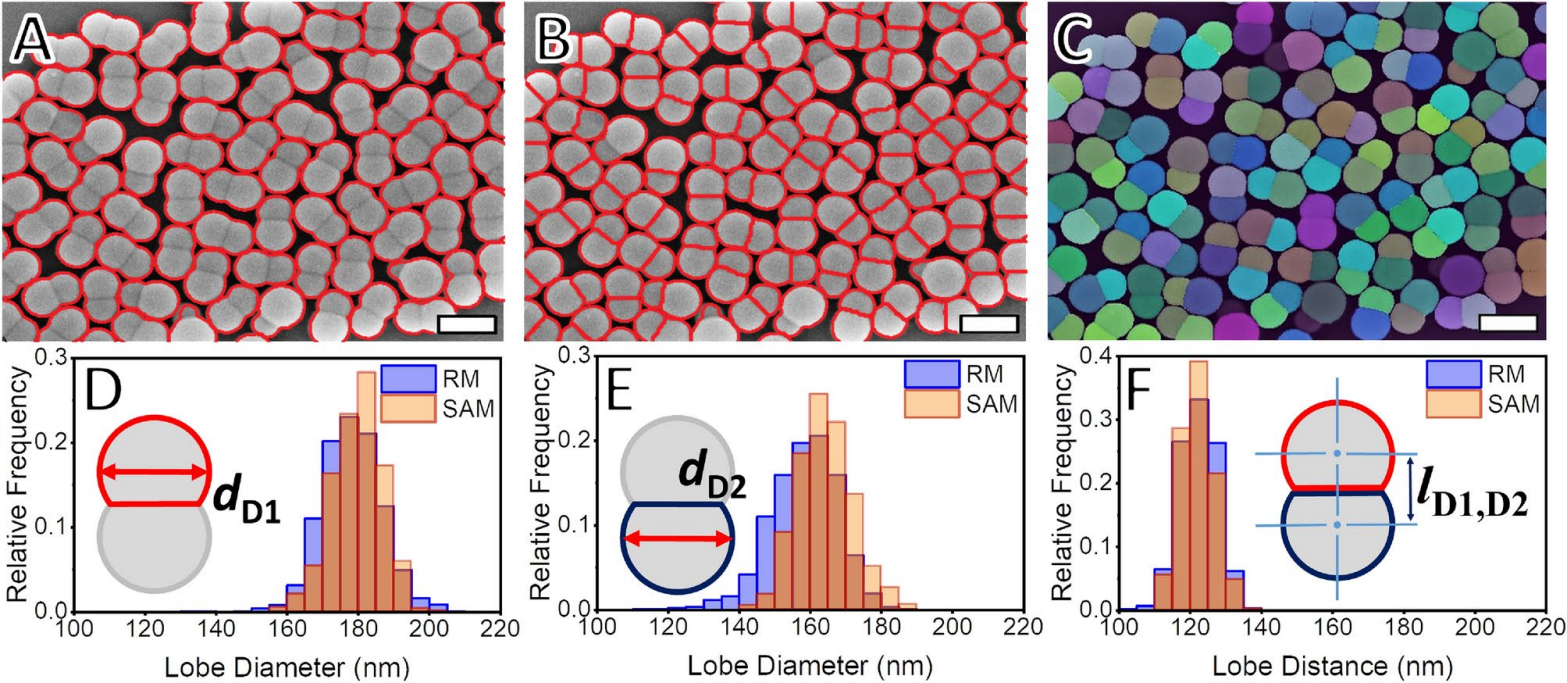
Comparison of the segmentation of dumbbell micrographs. Scale bars represent a length of 250 nm. (**A**) Low-sensitivity watershed segmentation identifies the dumbbells as a whole, and (**B**) high-sensitivity watershed segmentation identifies each of the dumbbell lobes individually. These two successive steps form the image segmentation in the reference method (RM) and are analogous to the one performed using the Segment Anything Model (SAM). (**C**) For the SAM, the segmentation directly differentiates between the individual lobes in each dumbbell. After the image segmentation is performed and the dumbbell lobes are assigned to the other lobes in the same particle, the size distributions for the (**D**) larger (*d*_D1_) and (**E**) smaller dumbbell lobes (*d*_D2_) can be determined. The distributions measured with RM and SAM are shown to have a high agreement. The same observation is made for the lobe distance distributions (*l*_D1D2_). The RM follows a methodology that is widely used in determining the morphology of particles^26,60^. The fact that the SAM method can deliver similar results can confirm this methodology as a viable option in determining the morphology of particle segments within complex arrangements. These results also prove that identifying the dumbbell lobes as part of larger assemblies is possible. With this, the SAM approach has proven its potential by pushing the limits and meeting the challenges in the morphology analysis of complex nanoparticles.

For the RM, the identification of the lobes starts with using the Euclidean-based watershed segmentation method with a low sensitivity or high tolerance (Fig. 3A). This step segments the images between the dumbbells without dividing between the lobes. The following step involves the same segmentation technique, but with a higher sensitivity or lower tolerance (Fig. 3B). In this case, the two-step image segmentation can discern between the dumbbells and their lobes. This process, however, can fail for up to 5% of the dumbbells, especially when one of the lobes is significantly smaller than the other one. This can be explained by the fact that the watershed methodology used in the RM is optimized for circular shapes^58,59^. One strategy to overcome the lack of segmentation between the dumbbell lobes could be using the watershed algorithm with a lower tolerance. However, this approach also leads to the segmentation processes taking place within the lobes and the consequent identification of a single lobe as two elements. In addition to the incapacity of the Watershed algorithm to segment between two highly overlapping circular projections, its use also demands a parameter-tuning process. With the SAM-based method, on the other hand, the image segmentation is correctly achieved using the same parameters employed for the spheres (Fig. 3C). The size distributions observed for the larger dumbbell lobes (*d*_D1_) (Fig. 3D) and for the smaller ones (*d*_D2_) (Fig. 3E) and for the lobe distance distribution (*l*_D1D2_) (Fig. 3F) show a high agreement between the measurements taken using the RM and the SAM. It can be noticed that the lobes segmented with the SAM method show a slightly higher diameter, following what is also observed for the spheres. The assignment of the dumbbell lobes was demonstrated by distinguishing between large and small lobes in the same particles and determining the distance between their centroids. This assignment has not been shown in similar works and represents a significant advancement over the most recent efforts to fully automate the morphological analysis of particles from micrographs^55^.

The comparison made on the basis of *d*_D1_, *d*_D2_ and *l*_D1D2_ and their corresponding distributions as obtained using the RM and the SAM are supported by their mean values and deviations (Table 2). The mean diameters of the larger dumbbell lobes *d*_D1,*n*_ differ by 1%, whereas the mean diameter of the smaller dumbbell lobes *d*_D2,*n*_ deviate by 4%. Despite the higher disagreement observed for *d*_D2,*n*_, the deviation is still within the experimental error expected for electron microscopy^17,18^. In both cases of *d*_D1,*n*_ and *d*_D2,*n*_ the PDI and SD values are lower when SAM is used instead of RM. The lower dispersity of the distributions observed here follows what was observed for the spheres. This could also be explained by the higher number of segmentation mistakes observed for RM. However, such mistakes don’t affect the distance between the dumbbell lobe centroids. This observation is based on the fact that the mean distance between the dumbbell lobes (*l*_D1D2,*n*_), diverges less than 1% between the two methods.

**Table 2:** Overview of morphological data obtained for the DiPS sample using both RM and SAM.

Measurement	Reference method			SAM-based method		
	Mean (nm)	SD (nm)	PDI	Mean (nm)	SD (nm)	PDI
Diameter large lobes D1, *d*_D1,*n*_	178	8	1.0023	180	7	1.0015
Diameter small lobes D2, *d*_D2,*n*_	158	10	1.0036	164	8	1.0022
Distance D1D2, *l*_D1D2,*n*_	122	5		124	9	

The values for the mean diameter of the large lobes (*d*_D1,*n*_), for the mean diameter of the small lobes (*d*_D2,*n*_), and for the mean distance between the lobes in each dumbbell (*l*_D1D2,*n*_) show a high level of agreement between the two methods. The same is also observed for the deviations in SD and PDI observed for the three analyzed distributions. The fact that very similar results are found when using either RM or SAM indicates that the latter can be used without detriment to the morphological analysis. This also confirms that it is possible to automatize the analysis of complex particles, including the determination of collective morphological properties.

### Analysis of trimer particles

The complexity of analzying trimer particles is even greater than that for the dumbbell particles. Here, the particles are composed of three lobes, and these must be individually identified and assigned to the trimers to which they belong. The three lobes are named T1 for the smaller trimer extremity, T2 for the lobe in the middle, and T3 for the larger extremity. Here, it must be made clear that T2 represents the lobe in the middle, not the one of medium size. Having T2 as the middle lobe means that its centroid is the vertex of the trimer angle ($\theta$). There may be an overlap between the size distributions of the lobes T1 and T2, meaning that the recognition of these lobes must be done using their relative positions in the form of the angle $\theta$. As they are not strictly the same from a chemical point of view, the distinction between them is essential for the chemical interpretation of the data^19^.

**Figure 4:**
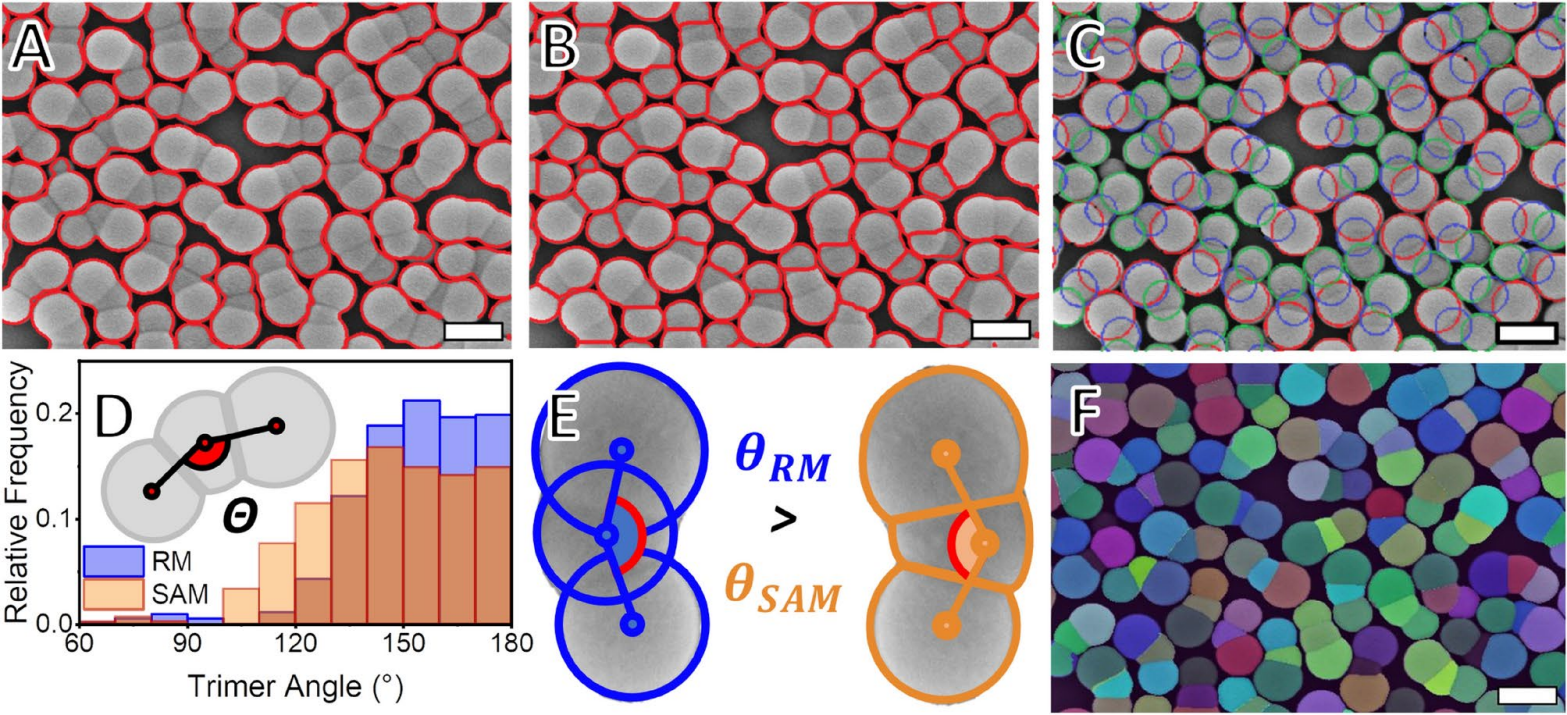
Comparison of different segmentation algorithms applied to the same image of the TriPS sample. (**A**) When the watershed algorithm is applied with low sensitivity to the trimer nanoparticles, it is possible to segment between them. (**B**) When the sensitivity is set to a higher value, however, the segmentation is not complete, and the lobes T2 and T3 are still identified as a single element. As the segmentation between T2 and T3 cannot be done solely using the watershed algorithm, the masks for each of the trimer lobes must be made manually (**C**). This makes the created masks overlap with each other, shifting the centroids from the trimer lobes and resulting in deviations in the trimer angle distribution (**D**,**E**). The shift, together with the unavoidable human bias present when the masks are manually prepared, urges for the development of a non-biased automatized method to characterize multi-lobe particles morphologically. The SAM segmentation of lobes is such a method (**F**) and succeeds in segmenting the lobes T2 and T3 from each other. The scale bars on the micrographs represent 250 nm.

Like for the two-lobed particles, a low-sensitivity watershed transformation is used to segment between the whole trimers (Fig. 4A). The next step in the RM workflow involves individually identifying the trimer lobes, a process that demands their segmentation from each other. This segmentation is similar to the one used for the dumbbells but not rigorously the same. The strategy to use a more sensitive watershed transformation on the second segmentation run fails to deliver consistent segmentation between T2 and T3 (see the Supplementary Information section, Fig. SI 1 and 2). The source of this problem is the fact that the watershed algorithm segments between elements based on their boundaries. The reliance on the boundaries of the elements leads to failures when discerning between overlapping regions^31^. The failure in segmenting between the projections of T2 and T3 reflects the high overlap between the circular projections of these lobes and confirms the limitations of the watershed segmentation method. These limitations led to the need to perform manual measurements to the RM to characterize T1, T2, and T3 individually. This approach was based on manually projecting ellipses over each of the lobes (Fig. 4C). This method takes around 10 h of labor to measure each set of images containing around 500 trimers and is subjected to human interference and bias^54^. The lengthy process of identifying the lobes using manual fitting and the inherent susceptibility to biased results highlight the need to develop a more efficient and accurate automated segmentation method.

The masking obtained using the SAM (Fig. 4F) shows how the segmentation between the lobes can be achieved with little to no trimers failing to be segmented. The first step in the morphological characterization is the identification of the lobes as elements of a particle. This process was conducted by minimizing the sum of distances between the centroids. The second step is determining the largest angle between the centroids of the three lobes present in a given set ($\theta$). The SAM segmentation of the trimer images, in contrast to the RM, made the determination of each lobe’s centroid using non-overlapping elements feasible. The methodological difference between RM and SAM in segmenting T1, T2, and T3 leads to measuring two different values for the same angle (Fig. 4D). This is observed with the trimer angle for the RM ($\theta$_RM_) being larger than the trimer angle measured for the SAM ($\theta$_SAM_). The methodological difference between the RM and the SAM when determining the centroid and the angle is illustrated in Fig. 4E. The determination of morphological aspects using centroids as references follows the common practice in modern simulations^12,61^.

**Figure 5:**
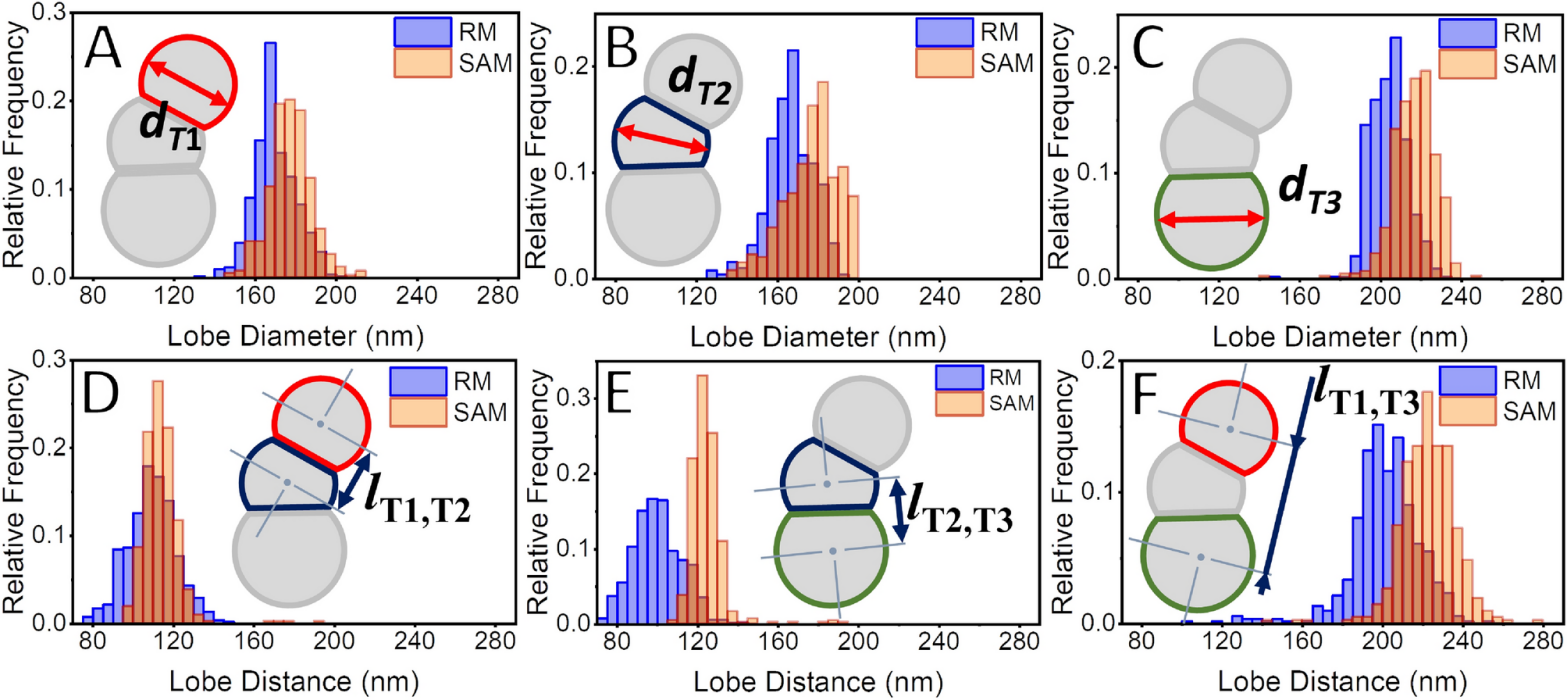
Lobe diameter distributions as measured for (**A**) T1 (*d*_T1,*n*_), (**B**) T2 (*d*_T2,*n*_), and (**C**) T3 (*d*_T3,*n*_) using both the manual RM and the automatized SAM. All the diameter distributions obtained using the RM are smaller than the ones obtained using the SAM, reflecting errors related to the overlap of the particles’ projections and possible human bias. The distributions of the distances between the lobes (**D**) T1 and T2 (*l*_T1,T2_), (**E**) T2 and T3 (*l*_T2,T3_) and (**F**) T1 and T3 (*l*_T1,T3_) also differ between RM and SAM. This latter discrepancy can be credited to the overlap between the lobes, which is not taken into consideration for the RM. This overlap between the masks in the RM measurements shifts the centroids of the lobes and leads to systemic error in the determination of the correct positioning of the trimers’ lobes and reflects on the distances between them.

It was shown that using the RM to segment the lobe projections on the images of TriPS requires manually fitting ellipses to each trimer lobe. This leads to errors arising from the overlap between the projections of the lobes when determining $\theta$ and the distance between the lobes (Fig. 5D–F). This contributes to the centroids of each lobe being identified in a different position from their expected location, thereby skewing the lobe distance distributions. The comparison of the RM to the SAM regarding the distance distribution between the lobes T1 and T2 (*l*_T1T2_) (Fig. 5D) shows a small discrepancy between the methods. The proximity between the *l*_T1T2_ measurements can be expected due to the low degree of overlap between the projections of the lobes T1 and T2. As the overlap between the ellipses fitted to T1 and T2 is low, the shift in the relative positions of the lobes is also low. For the distance between the lobes T2 and T3 (*l*_T2T3_) (Fig. 5E), however, the discrepancy between the distributions obtained with the RM and with the SAM is larger. The larger disagreement is seen due to the larger degree of overlap between the projections of T2 and T3. As the projection overlap is high, so is the shift in the relative centroid position of the masks used in RM. The shift in the relative positions of the centroids of T2 and T3 when RM is used explains the discrepancy between (*l*_T2T3_) measured using RM and SAM. When the distance distributions between the lobes T1 and T3 (*l*_T1T3_) are observed (Fig. 5F), a disagreement between the RM and the SAM is found. The overlap of the projections of T1 and T3 with the projection of T2 for RM can explain this finding. The discrepancy between the lobe distance data gathered using SAM and RM once more corroborates the efficacy of employing a non-overlapping masking technique in the form of SAM to morphologically characterize particles from micrographs. Additionally, the manual fitting of masks can lead to errors regarding the size distribution of the lobes^23,54^. This assumption is supported by the size distributions observed for all the trimer lobes (Fig. 5). It can be seen that the diameter distributions of T1 (*d*_T1_) (Fig. 5A), T2 (*d*_T2_) (Fig. 5b), and T3 (*d*_T3_) (Fig. 5C) are different when measured using RM or SAM.

It was observed that the values determined for $\theta$, inter-lobe distances, and lobe sizes differ between BM and SAM. This observation was taken from the distributions presented in Figs. 4 and. 5 and shown in detail in Table 3. Part of the error associated with measuring the individual lobes manually can be attributed to human bias when creating the masks for these individual trimer lobes^23,54^. Additionally, RM itself is credited as a source of systemic error due to the nature of the overlapping projections of each trimer lobe. Because of that, the results would still fail to indicate the correct positions of each lobe, even with a perfect manual fit. This positioning error is unavoidable when circles are projected over lobes made up of overlapping spheres. This underlines the need to use segmentation methodologies capable of creating masks for complex nanoparticles without overlapping labels. The measurement of collective morphological properties like the lobe distances and the trimer angle confirm that the masks identifying the trimer lobes can be assigned to each other. This assignment represents an advancement to what is currently present in the literature for similar works.

**Table 3:** Overview of morphological data obtained for the TriPS sample using both the RM and SAM.

Measurement	Reference method			SAM-based method		
	Mean (nm)	SD (nm)	PDI	Mean (nm)	SD (nm)	PDI
Diameter trimer lobe T1, *d*_T1,*n*_	170	10	1.0033	178	8	1.0033
Diameter trimer lobe T2, *d*_T2,*n*_	168	11	1.0044	178	14	1.0063
Diameter trimer lobe T3, *d*_T3,*n*_	204	9	1.0018	216	11	1.0023
Distance T1T2, *l*_T1,T2, n_	109	12		114	9	
Distance T2T3, *l*_T2,T3, n_	100	13		124	9	
Distance T1T3, *l*_T1,T3, n_	201	18		224	16	
	Mean ($^\circ$)	SD ($^\circ$)		Mean ($^\circ$)	SD ($^\circ$)	
Trimer angle, $\theta$_n_	154	19		146	22	

The deviations in the values between the values obtained using the RM and the SAM point to the fact that the data collection for the RM is not ideal. RM demands that overlapping projections to be fitted to the trimer lobes manually. This effectively works as a source of systemic error and explains the disagreement in the values observed between RM and SAM.

## Conclusion

The study presented a novel method that uses a pre-trained deep learning model to automate the segmentation and morphological characterization of nanoparticles with varying degree of complexity from micrographs, showing higher accuracy and faster analysis than traditional methods. The deep learning model, known as the Segment Anything Model (SAM) had already been undergone generalist training. The stage that required the longest time, namely the model training, is therefore no longer necessary. This significantly reduces the time required for the entire analysis, making it much faster than manual measurements or dedicated deep-learning models.

A comparison of the results obtained using the SAM model with those obtained through a traditional approach allowed for the validation of the data and demonstrated that the acceleration of the analysis did not result in any loss of accuracy. The novel method thus avoids the need for labor-intensive manual measurements The comparison further demonstrated that the SAM-based method presented here is effective in segmenting between instances where the traditional erosion and dilation-based methods fail, offering an accurate and reliable approach for analyzing complex nanoparticles.

This is particularly important in the case of particles composed of segments with highly overlapping circular projections. The handling of partial overlap between individual segments within segmented particles or particle aggregates represents a significant challenge in the field of image segmentation. The evaluation of pre-trained segmentation models with particle dimers and trimers has revealed the distinctive capabilities of the SAM models in addressing this challenge. This is achieved through the identification of labels derived from the overlap of multiple masks, enabling the appropriate treatment of these complex structures. The appropriate assignment of subdivisions within a complex particle is made in accordance with the particle to which they belong, facilitating the extraction of more detailed information from a single image. This enables the utilization of the method even in instances where a meticulous determination of *ground truth* is unfeasible, despite the most rigorous manual evaluation, due to the superimposition of neighboring structural elements. In light of the methodology that has been developed thus far, a future direction is to expand the method for compositional analyses of samples comprising different sets of particles. It is hypothesized that this advancement could be achieved through post-processing utilizing the masks generated by our method. This would facilitate new avenues in the investigation of aggregation processes that are of practical relevance in diverse fields. Furthermore, it is anticipated that the method can be expanded to other structures and systems, extending beyond the fields of colloidal particles and microscopy.

## Supplementary Information

Supplementary Information.

## Data Availability

Complete experimental data and developed codes are accessible via the institutional server registered with the DOI 10.48606/EsfTYSZxEqPwiVkZ. All accession codes are available via GitHub.

## References

[1] Jones, R. G. et al. *Compendium of Polymer Terminology and Nomenclature* (International Union of Pure and Applied Chemistry, Cambridge, 2009).

[2] Plüisch, C.S. & Wittemann, A. Assembly of nanoparticles into “colloidal molecules”: Toward complex and yet defined colloids with exiting perspectives. *Adv. Colloid Sci.* 237–264. 10.5772/65343 (2016).

[3] Yuan, W. et al. Structural color fibers directly drawn from colloidal suspensions with controllable optical properties. *ACS Appl. Mater. Interfaces.* **5**, 19388–19396. 10.1021/acsami.8b21070 (2019). 10.1021/acsami.8b21070 31067026

[4] Nishiguchi, A., Kurihara, Y. & Taguchi, T. Hemostatic, tissue-adhesive colloidal wound dressing functionalized by uv irradiation. *ACS Appl. Bio Mater.* **3**, 1705–1711. 10.1021/acsabm.0c00015 (2020). 35021659 10.1021/acsabm.0c00015

[5] Liu, G. et al. Hairy polyelectrolyte brushes-grafted thermosensitive microgels as artificial synovial fluid for simultaneous biomimetic lubrication and arthritis treatment. *ACS Appl. Mater. Interfaces.* **6**, 20452–20463. 10.1021/am506026e (2014). 25347384 10.1021/am506026e

[6] Tzounis, L., Doña, M., Lopez-Romero, J., Fery, A. & Contreras-Caceres, R. Temperature-controlled catalysis by core-shell-satellite auag@pnipam@ag hybrid microgels: A highly efficient catalytic thermoresponsive nanoreactor. *ACS Appl. Mater. Interfaces.* **11**, 29360–29372. 10.1021/acsami.9b10773 (2019). 31329406 10.1021/acsami.9b10773

[7] Sabadasch, V. et al. Pd Nanoparticle-Loaded Smart Microgel-Based Membranes as Reusable Catalysts. *ACS Appl. Mater. Interfaces.* **14**, 49181–49188. 10.1021/acsami.2c14415 (2022). 36256601 10.1021/acsami.2c14415

[8] Zhang, Q., Zeng, S., Lin, B. & Qin, J. Controllable synthesis of anisotropic elongated particles using microvalve actuated microfluidic approach. *J. Mater. Chem.* **21**, 2466–2469. 10.1039/c0jm04033a (2011).

[9] Wang, C., Xie, Q., Wang, Y. & Fu, L. Effect of ultrasound treatment on allergenicity reduction of milk casein via colloid formation. *J. Agric. Food Chem.* **68**, 4678–4686. 10.1021/acs.jafc.9b08245 (2020). 32274927 10.1021/acs.jafc.9b08245

[10] Stuckert, R., Plüisch, C. S. & Wittemann, A. Experimental assessment and model validation on how shape determines sedimentation and diffusion of colloidal particles. *Langmuir* **34**, 13339–13351. 10.1021/acs.langmuir.8b02999 (2018). 30350686 10.1021/acs.langmuir.8b02999

[11] Forster, J. et al. Assembly of optical-scale dumbbells into dense photonic crystals. *ACS Nano* **5**, 6695–6700. 10.1021/nn202227f (2011). 21740047 10.1021/nn202227f

[12] Stuckert, R., Lüders, A., Wittemann, A. & Nielaba, P. Phase behaviour in 2d assemblies of dumbbell-shaped colloids generated under geometrical confinement. *Soft Matter* **17**, 6519–6535. 10.1039/D1SM00635E (2021). 34180929 10.1039/d1sm00635e

[13] Sturm, E. V. & Cölfen, H. Mesocrystals: Past, presence, future. *Curr. Comput.-Aided Drug Des.* **7**, 207. 10.3390/cryst7070207 (2017).

[14] Plüisch, C. S., Stuckert, R. & Wittemann, A. Direct measurement of sedimentation coefficient distributions in multimodal nanoparticle mixtures. *Nanomaterials* **11**, 1027–1045. 10.3390/nano11041027 (2021). 33920635 10.3390/nano11041027 PMC8072784

[15] Plüisch, C. S. & Wittemann, A. Shape-tailored polymer colloids on the road to become structural motifs for hierarchically organized materials. *Macromol. Rapid Commun.* **34**, 1798–1814. 10.1002/marc.201300693 (2013). 24327380 10.1002/marc.201300693

[16] Bergmann, S., Wrede, O., Huser, T. & Hellweg, T. Super-resolution optical microscopy resolves network morphology of smart colloidal microgels. *Phys. Chem. Chem. Phys.* **20**, 5074–5083. 10.1039/C7CP07648G (2018). 29392265 10.1039/c7cp07648g

[17] Crouzier, L. et al. Methodology to evaluate the uncertainty associated with nanoparticle dimensional measurements by sem. *Meas. Sci. Technol.* **30**, 085004. 10.1088/1361-6501/ab1495 (2019).

[18] Eaton, P. et al. A direct comparison of experimental methods to measure dimensions of synthetic nanoparticles. *Ultramicroscopy* **182**, 179–190. 10.1016/j.ultramic.2017.07.001 (2017). 28692935 10.1016/j.ultramic.2017.07.001

[19] Monteiro, G. & Wittemann, A. Linear growth of colloidal dumbbells into three-lobed polymer nanoparticles mediated by a gradient in surface wettability. *Colloid Polym. Sci.* **301**, 801–812. 10.1007/s00396-023-05131-z (2023).

[20] Smits, H. J. et al. Validation of automated positive cell and region detection of immunohistochemically stained laryngeal tumor tissue using digital image analysis. *J. Pathol. Inf.* **14**, 100198. 10.1016/j.jpi.2023.100198 (2023). 10.1016/j.jpi.2023.100198 PMC9930147 36818021

[21] Aboy-Pardal, M. C. et al. A deep learning-based tool for the automated detection and analysis of caveolae in transmission electron microscopy images. *Comput. Struct. Biotechnol. J.* **21**, 224–237. 10.1016/j.csbj.2022.11.062 (2023). 36544477 10.1016/j.csbj.2022.11.062 PMC9755247

[22] Yang, B. et al. Following nanoparticle uptake by cells using high-throughput microscopy and the deep-learning based cell identification algorithm cellpose. *Front. Nanotechnol.* **5**. 10.3389/fnano.2023.1181362 (2023).

[23] Li, W., Field, K.G. & Morgan, D. Automated defect analysis in electron microscopic images. *NPJ Comput. Mater.* **4**. 10.1038/s41524-018-0093-8 (2018).

[24] Schulz, B., Haghdadi, N., Leitner, T., Hafok, M. & Primig, S. Advancing analytical electron microscopy methodologies to characterise microstructural features in superalloys. *Ultramicroscopy* **247**, 113699. 10.1016/j.ultramic.2023.113699 (2023). 36753846 10.1016/j.ultramic.2023.113699

[25] De Temmerman, P.-J., Verleysen, E., Lammertyn, J. & Mast, J. Semi-automatic size measurement of primary particles in aggregated nanomaterials by transmission electron microscopy. *Powder Technol.* **261**, 191–200. 10.1016/j.powtec.2014.04.040 (2014).

[26] Rühle, B., Krurmey, J. F. & Hodoroba, V.-D. Workflow towards automated segmentation of agglomerated, non-spherical particles from electron microscopy images using artificial neural networks. *Sci. Rep.* **18**, 529. 10.1186/s12859-017-1934-z (2017). 10.1038/s41598-021-84287-6 PMC7925552 33654161

[27] Yildirim, B. & Cole, J. M. Bayesian particle instance segmentation for electron microscopy image quantification. *J. Chem. Inf. Model.* **61**, 1136–1149. 10.1021/acs.jcim.0c01455 (2021). 33682402 10.1021/acs.jcim.0c01455 PMC8041280

[28] Rueden, C. T. et al. ImageJ 2: ImageJ for the next generation of scientific image data. *Sci. Rep.* **11**, 4942. 10.1038/s41598-021-84287-6 (2021). 33654161 10.1038/s41598-021-84287-6 PMC7925552

[29] Kim, H., Han, J. & Han, T. Y. J. Hairy polyelectrolyte brushes-grafted thermosensitive microgels as artificial synovial fluid for simultaneous biomimetic lubrication and arthritis treatment. *Nanoscale* **12**, 19461–19469. 10.1039/d0nr04140h (2020). 25347384 10.1021/am506026e

[30] Faraz, K., Grenier, T., Ducottet, C. & Epicier, T. Deep learning detection of nanoparticles and multiple object tracking of their dynamic evolution during in situ etem studies. *Sci. Rep.* **12**. 10.1038/s41598-022-06308-2 (2022). 10.1038/s41598-022-06308-2 PMC8847623 35169206

[31] Xu, H., Xing, Y. & Wang, W. Image segmentation with boundary-to-pixel direction and magnitude based on watershed and attention mechanism. *SIViP* **17**, 1695–1703. 10.1007/s11760-022-02380-3 (2023).

[32] LeCun, Y., Bengio, Y. & Hinton, G. Deep learning. *Nature* **521**, 436–44. 10.1038/nature14539 (2015). 26017442 10.1038/nature14539

[33] Lateef, F. & Ruichek, Y. Survey on semantic segmentation using deep learning techniques. *Neurocomputing* **338**, 321–348. 10.1016/j.neucom.2019.02.003 (2019).

[34] Otsu, N. A threshold selection method from gray-level histograms. *IEEE Trans. Syst. Man Cybern.* **9**, 62–66. 10.1109/TSMC.1979.4310076 (1979).

[35] Neubert, P. & Protzel, P. Compact watershed and preemptive slic: On improving trade-offs of superpixel segmentation algorithms. In *2014 22nd international conference on pattern recognition*, 996–1001. 10.1109/ICPR.2014.181 (IEEE, 2014).

[36] Vedaldi, A. & Soatto, S. Quick shift and kernel methods for mode seeking. In *Computer Vision–ECCV 2008: 10th European Conference on Computer Vision, Marseille, France, October 12-18, 2008, Proceedings, Part IV 10*, 705–718, 10.1007/978-3-540-88693-8_52 (Springer, 2008).

[37] Achanta, R. et al. Slic superpixels compared to state-of-the-art superpixel methods. *IEEE Trans. Pattern Anal. Mach. Intell.* **34**, 2274–2282. 10.1109/TPAMI.2012.120 (2012). 22641706 10.1109/TPAMI.2012.120

[38] Breiman, L. *Random forests. Machine learning* **45**, 5–32. 10.1023/A:1010933404324 (2001).

[39] Matskevych, A., Wolny, A., Pape, C. & Kreshuk, A. From shallow to deep: exploiting feature-based classifiers for domain adaptation in semantic segmentation. *Front. Comput. Sci.* **4**, 805166. 10.3389/fcomp.2022.805166 (2022).

[40] Cates, J. E., Whitaker, R. T. & Jones, G. M. Case study: An evaluation of user-assisted hierarchical watershed segmentation. *Med. Image Anal.* **9**, 566–578. 10.1016/j.media.2005.04.007 (2005). 15919233 10.1016/j.media.2005.04.007

[41] Szegedy, C. et al. Going deeper with convolutions. In *Proceedings of the IEEE conference on computer vision and pattern recognition*, 1–9. 10.1109/CVPR.2015.7298594 (2015).

[42] He, K., Gkioxari, G., Dollár, P. & Girshick, R. Mask r-cnn. In *Proceedings of the IEEE international conference on computer vision*, 2961–2969. 10.1109/ICCV.2017.322 (2017).

[43] Ronneberger, O., Fischer, P. & Brox, T. U-net: Convolutional networks for biomedical image segmentation. In *Medical Image Computing and Computer-Assisted Intervention–MICCAI 2015: 18th International Conference, Munich, Germany, October 5-9, 2015, Proceedings, Part III 18*, 234–241, 10.48550/arXiv.1505.04597 (Springer, 2015).

[44] Badrinarayanan, V., Kendall, A. & Cipolla, R. Segnet: A deep convolutional encoder-decoder architecture for image segmentation. *IEEE Trans. Pattern Anal. Mach. Intell.* **39**, 2481–2495. 10.1109/TPAMI.2016.2644615 (2017). 28060704 10.1109/TPAMI.2016.2644615

[45] Prajna, Y. & Nath, M. K. A survey of semantic segmentation on biomedical images using deep learning. In Harvey, D., Kar, H., Verma, S. & Bhadauria, V. (eds.) *Advances in VLSI, Communication, and Signal Processing*, 347–357, 10.1007/978-981-15-6840-4_27 (Springer Singapore, Singapore, 2021).

[46] Li, B., Liu, S., Xu, W. & Qiu, W. Real-time object detection and semantic segmentation for autonomous driving. In *MIPPR 2017: Automatic Target Recognition and Navigation*, vol. 10608, 167–174, 10.1117/12.2288713 (SPIE, 2018).

[47] Li, W., Chen, H. & Shi, Z. Semantic segmentation of remote sensing images with self-supervised multitask representation learning. *IEEE J. Select. Topics Appl. Earth Observ. Remote Sens.*[SPACE]10.1109/JSTARS.2021.3090418 (2021).

[48] Monteiro, B. A. A., Oliveira, H. & Santos, J. A. Self-supervised learning for seismic image segmentation from few-labeled samples. *IEEE Geosci. Remote Sens. Lett.* **19**, 1–5. 10.1109/LGRS.2022.3193567 (2022).

[49] Jing, L. & Tian, Y. Self-supervised visual feature learning with deep neural networks: A survey. *IEEE Trans. Pattern Anal. Mach. Intell.* **43**. 10.48550/arXiv.1902.06162 (2019). 10.1109/TPAMI.2020.2992393 32386141

[50] Su, J.C., Maji, S. & Hariharan, B. When does self-supervision improve few-shot learning? *Lecture Notes in Computer Science (including subseries Lecture Notes in Artificial Intelligence and Lecture Notes in Bioinformatics)* **12352 LNCS**, 645–666, 10.1007/978-3-030-58571-6_38 (2020).

[51] Sun, Q., Liu, Y., Chua, T.-S. & Schiele, B. Meta-transfer learning for few-shot learning. In *CVPR*, 403–412. 10.48550/arXiv.1812.02391 (2019).

[52] Xian, Y., Lampert, C. H., Schiele, B. & Akata, Z. Zero-shot learning – a comprehensive evaluation of the good, the bad and the ugly, 10.48550/arXiv.1707.00600 (2020). 10.1109/TPAMI.2018.2857768 30028691

[53] Kirilov, A. et al. Segment anything. *arXiv*[SPACE]10.48550/arXiv.2304.02643 (2023).

[54] Bruno, G., Lynch, M. J., Jacobs, R., Morgan, D. D. & Field, K. G. Evaluation of human-bias in labeling of ambiguous features in electron microscopy machine learning models. *Microsc. Microanal.* **29**, 1493–1494. 10.1093/micmic/ozad067.767 (2023).

[55] Larsen, R., Villadsen, T.L., Mathiesen, J.K., Jensen, K.M. & Boejesen, E.D. Np-sam: Implementing the segment anything model for easy nanoparticle segmentation in electron microscopy images. *ChemRxiv*[SPACE]10.26434/chemrxiv-2023-k73qz-v2 (2023).

[56] Arena, E.T. et al. Quantitating the cell: turning images into numbers with ImageJ. *Wiley Interdiscip. Rev. Dev. Biol.* **6**, 10.1002/wdev.260 (2017). 10.1002/wdev.260 27911038

[57] van der Walt, S. et al. scikit-image: image processing in python. *PeerJ* **2**, e453. 10.7717/peerj.453 (2014). 25024921 10.7717/peerj.453 PMC4081273

[58] Watershed separation. *ImageJ Pugin*[SPACE]https://imagej.net/imaging/watershed (2022). [Online; accessed 01-September-2023].

[59] Schmid, M. Adjustable watershed. *ImageJ Pugin*[SPACE]https://imagej.net/plugins/adjustable-watershed/adjustable-watershed (2022). [Online; accessed 01-September-2023].

[60] Mazzoli, A. & Favoni, O. Particle size, size distribution and morphological evaluation of airborne dust particles of diverse woods by scanning electron microscopy and image processing program. *Powder Technol.* **225**, 65–71. 10.1016/j.powtec.2012.03.033 (2012).

[61] Heptner, N. & Dzubiella, J. Equilibrium structure and fluctuations of suspensions of colloidal dumbbells. *Mol. Phys.* **113**, 2523–2530. 10.1080/00268976.2015.1022609 (2015).

