# Supplementary material for: Pre-trained artificial intelligence-aided analysis of nanoparticles using the segment anything model
Source: Sci Rep. 2025 Jan 17;15:2341. doi: 10.1038/s41598-025-86327-x (PMC11748653; doi:10.1038/s41598-025-86327-x)
Supplement: Supplementary file 1 — Supplementary Information. [file 41598_2025_86327_MOESM1_ESM.pdf]

SUPPLEMENTARY INFORMATION
Pre-Trained Artificial Intelligence-Aided Analysis of Nanoparticles Using the Segment Anything Model

Gabriel A. A. Monteiro[1,+], **Bruno A. A. Monteiro**[2,+], **Jefersson A. dos Santos**[2,3,*], **and Alexander Wittemann**[1,*]

[1]Colloid Chemistry, Department of Chemistry, University of Konstanz, Universitaetsstrasse 10, Konstanz, D-78464, Germany
[2]Pattern Recognition and Earth Observation Laboratory, Department of Computer Science, Federal University of Minas Gerais, Belo Horizonte, 31270-901, Brazil
[3]Department of Computer Science, University of Sheffield, S1 4DP, Sheffield, United Kingdom
[*]alexander.wittemann@uni-konstanz.de, j.santos@sheffield.ac.uk
[+]these authors contributed equally to this work

Limitations of the *Watershed* algorithm in segmenting highly overlapping lobes in comparison to the Segment Anything Method

The Segment Anything Model (SAM) was originally trained using a wide array of images and elements present in these images[1]. The broadness of the training phase allows its use in several fields, including identifying particles in micrographs[2]. Alongside SAM, the *Watershed* algorithm can also be used in particle characterization[3], but the latter still has some drawbacks[2,4,5]. These drawbacks are especially amplified when the particles are not spherical[5], are in close contact, or appear to overlap in the micrograph[2]. The trimer particles (TriPS) have a morphology that could amplify the drawbacks from *Watershed*.

A series of experiments were conducted to check whether the *Watershed* algorithm in the reference method (RM) could correctly segment between the trimer lobes. These experiments were based on varying the value of the tolerance of the *Watershed* ImageJ plugin and on applying morphological constraints to the masks obtained. In Fig. SI 1 the relative amount of masks found applying the *Watershed* algorithm to the TriPS images is presented. The number of masks is presented in comparison to the number of lobes counted manually in the TriPS images ("Manual" line), to the number of masks found using SAM ("SAM" line) and to the number of masks using SAM morphological constraints ("SAM, Morphological Constraints" line). The relative number of elements found is presented as a function of the *Watershed* tolerance and sensitivity, where sensitivity = tolerance^{-1}. The morphological constraints are applied to discard segmented instances that do not represent particles or lobes but parts of them or other artefacts. The morphological constraints imposed on the masks obtained with SAM and *Watershed* were the same. The number of elements found with either *Watershed* or SAM was normalized to the number found when counting the trimer lobes manually. The error bars represent the standard deviation in the relative number of masks found for the different analyzed images. This relative number does not necessarily mean that the masks correctly represent the trimer lobes in the sample. It only represents the number of masks. It is found that the number of masks found with *Watershed* is the lowest for high tolerance values. High tolerance values represent a higher distance between mask centroids, explaining the lower density of masks found in this case[5]. For lower tolerance values, the amount of masks becomes larger, reflecting the larger number of segmentation lines formed by the *Watershed* algorithm. Throughout all of the range of *Watershed* tolerance values, the relative number of masks found without constraints (blue curve with squares, from 0.4 ($\pm$ 0.1) to 1.2 ($\pm$ 0.2)) is higher than the number of masks found when the constraints are imposed (red curve with circles, from 0.32 ($\pm$ 0.02) to 0.89 ($\pm$ 0.04)). This means that some of the masks found using *Watershed* were not accurate in describing the projection area of a potential particle or lobe. As they were inaccurate, they are removed before further analysis using RM. An important result here is the relative amount of masks found when using *Watershed* with the highest tolerance and with the morphological constraints. In this case, 32 $\pm$ 2 masks were found for every 100 lobes in the image. This value represents the number of trimers per lobe, 1/3, and indicates that the segmentation between trimer particles used in the RM was accurate. The number of masks found using SAM was the highest among all of the cases analyzed ("SAM" horizontal line, 1.64 ($\pm$ 0.04)). SAM is optimized to find as many segments as possible, even identifying background areas as masks. Another difference between SAM and *Watershed* is that SAM allows for overlapping masks and *Watershed* does not. This means some SAM masks represent the same area in the image[1]. One example would be two neighboring lobes being described by one mask each and a third mask simultaneously representing both. With that, it can be expected that SAM finds more elements in an image than the *Watershed* and even more

than there really are. Some of these extra SAM masks are removed using morphological constraints ("SAM Morphological Constraints" horizontal line, 1.19 ($\pm$ 0.07)), but this method still finds more masks than there are lobes in an image. This happens because the combined area of the smaller trimer lobes (T1 and T2) is similar to that of the largest one (T3). With that, the separation between masks that represent T1 and T2 and masks that represent T3 is impossible solely using their size as a comparison parameter.

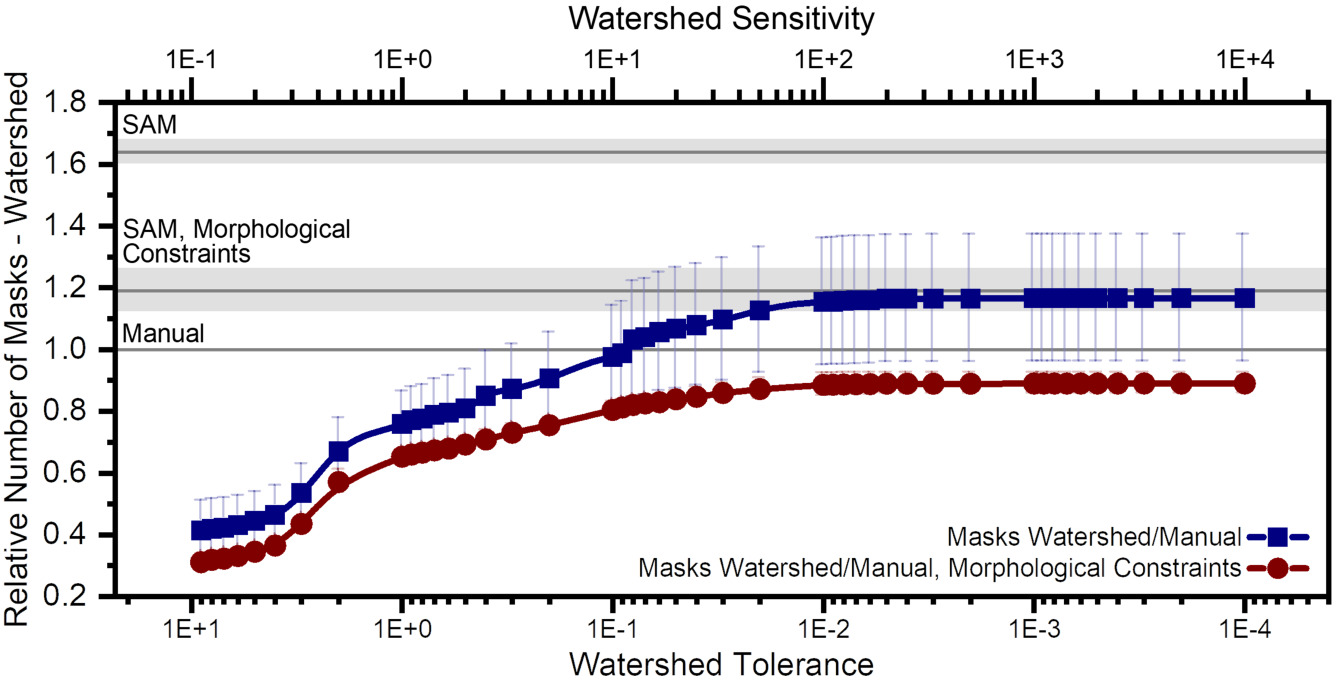

Figure SI 1. Normalized number of masks found using *Watershed* and SAM. The measurements are normalized to the number of trimers found when manually counting the particles. Both are presented with and without morphological constraints to the identified masks. Masks obtained using *Watershed* are shown as a function of the tolerance value and are represented by a curve. The number of masks observed with SAM are shown as lines, reflecting that no parameters were changed during this analysis. The amount of masks seen with SAM is much higher than the number observed with *Watershed*. This reflects the fact that SAM identifies overlapping masks. The fact that the SAM masks overlap results in this technique identifying more masks than there are TriPS lobes in the image. The morphological constraints in both the SAM and *Watershed* lower the number of identified masks. This lower number is seen because some of the masks obtained do not accurately describe particles or particle lobes and are discarded before further analysis.

Examples of the masks obtained using *Watershed* on the TriPS samples are presented in Fig. SI 2 alongside the corresponding region of the micrograph for which the masks were obtained. For high tolerance values (10), the trimer images were segmented from each other, but no segmentation within the projection of each particle was observed. This corroborates the idea presented for Fig. SI 1 that the *Watershed* algorithm is accurate in segmenting between the trimers but not within them. When lower *Watershed* tolerance values are used, some segmentation between the lobes begins to be observed. This segmentation happens predominantly between the lobes T1 and T2, the smaller ones. Their overlap is lower than that between T2 and T3[3] (see Fig. 5), explaining the preferential segmentation[5]. The number of masks found for tolerance values between 10^{-2} and 10^{-4} remains roughly the same. This corroborates Fig. SI 1, where the number of masks remains roughly constant in the 10^{-2} to 10^{-4} interval. Setting morphological constraints causes some masks to be ignored and not shown in Fig. SI 2. When lower *Watershed* tolerance values are used, more segmentation lines are formed close to each other. This creates small masks between these lines. They are too small for the employed morphological constraints, so they are not considered for further analysis. This is more prominent between the TriPS lobes T1 and T2 and for tolerance values lower than 10^{-2}.

Overall, the analysis of the masks obtained with the *Watershed* plugin shows the inaccuracy of this approach when segmenting between highly overlapping elements. Although the number of masks found with *Watershed* might be close to the number of TriPS lobes present, the spacial accuracy is still lacking. This shows that the number of masks should not be used as a basis for comparison to determine the accuracy of the segmentation methodology.

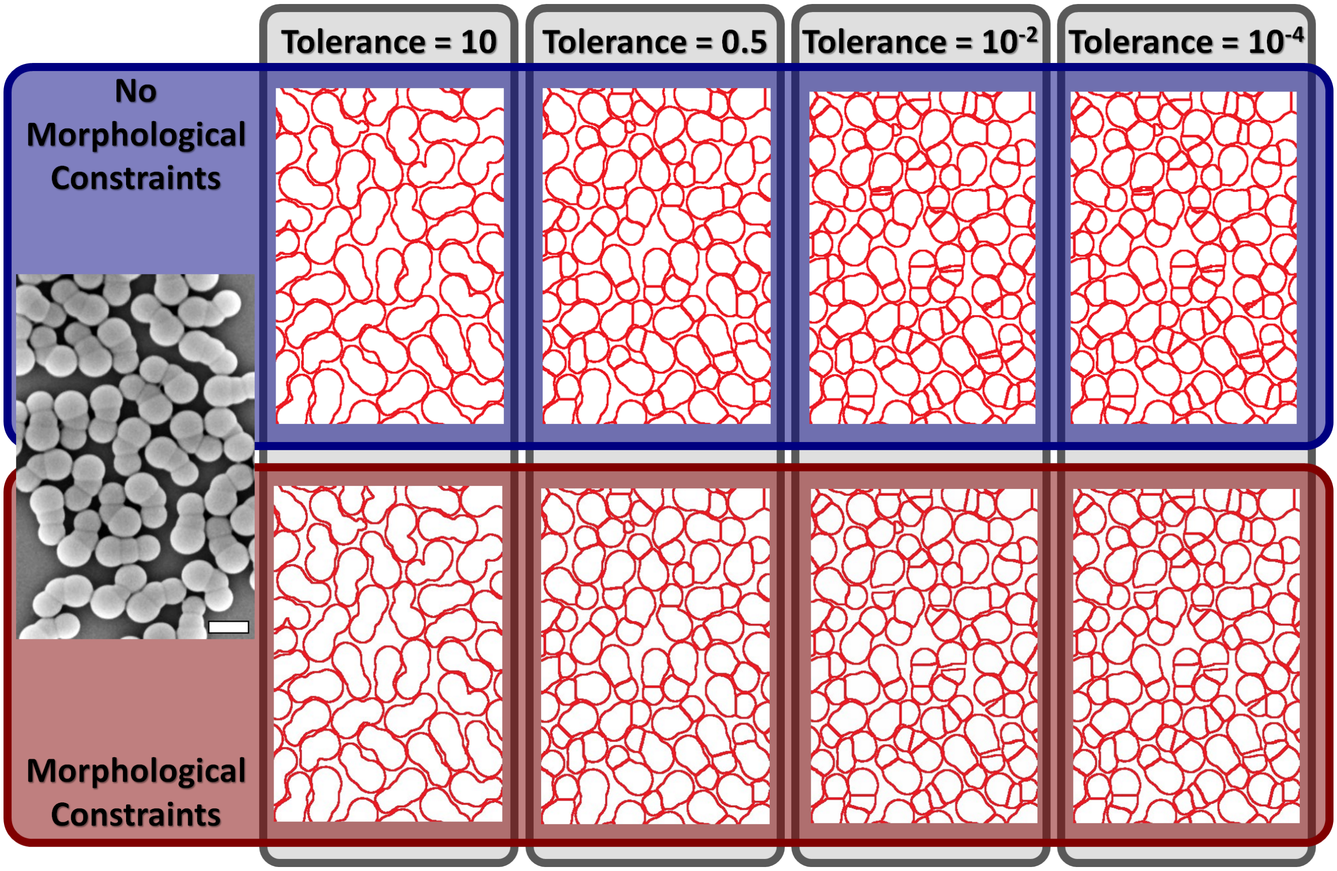

Figure SI 2. Segmentation of TriPS micrographs obtained with the *Watershed* ImageJ plugin with tolerance values ranging from 10 to 10^{-4} including the standard *Watershed* value of 0.5. The masks obtained are presented with and without morphological constraints. The micrograph section from which these masks were derived is shown for the sake of comparison. It can be seen that a filter with a high tolerance level of 10 can accurately segment between the trimer particles. This segmentation is absent between the lobes in each particle. This indicates the accuracy of the *Watershed* algorithm in segmenting between the particles as a whole. However, multiple segmentation lines are observed for lower tolerance values, mostly between the trimer lobes T1 and T2. At the same time, the segmentation between T2 and T3 is rare. This discrepancy reflects the difference in the overlapping degrees between T1 and T2 and between T2 and T3 (see Fig. 5). Small areas are formed between segmentation lines, especially between T1 and T2 and when low tolerance values are used. The morphological constraints ignore these and corroborate Fig. SI 1. The scale bar on the SEM image represents 250 nm.

References

1. Kirilov, A. *et al.* Segment anything. *arXiv* https://doi.org/10.48550/arXiv.2304.02643 (2023).
2. Larsen, R., Villadsen, T. L., Mathiesen, J. K., Jensen, K. M. & Boejesen, E. D. Np-sam: Implementing the segment anything model for easy nanoparticle segmentation in electron microscopy images. *ChemRxiv* DOI: 10.26434/chemrxiv-2023-k73qz-v2 (2023).
3. Monteiro, G. & Wittemann, A. Linear growth of colloidal dumbbells into three-lobed polymer nanoparticles mediated by a gradient in surface wettability. *Colloid Polym. Sci.* **301**, 801–812, DOI: https://doi.org/10.1007/s00396-023-05131-z (2023).
4. Xu, H., Xing, Y. & Wang, W. Image segmentation with boundary-to-pixel direction and magnitude based on watershed and attention mechanism. *Signal, Image Video Process.* **17**, 1695–1703, DOI: https://doi.org/10.1007/s11760-022-02380-3 (2023).
5. Schmid, M. Adjustable watershed. *ImageJ Pugin* https://imagej.net/plugins/adjustable-watershed/adjustable-watershed (2022). [Online; accessed 01-September-2023].
